# Pure Social Disparities in Distribution of Dentists: A Cross-Sectional Province-Based Study in Iran

**DOI:** 10.3390/ijerph10051882

**Published:** 2013-05-06

**Authors:** Aliasghar A. Kiadaliri, Reza Hosseinpour, Hassan Haghparast-Bidgoli, Ulf-G Gerdtham

**Affiliations:** 1 Division of Health Economics, Department of Clinical Sciences-Malmö, Lund University, Malmö 20502, Sweden; E-Mail: ulf.gerdtham@med.lu.se; 2 Department of Health Management and Economics, School of Public Health, Tehran University of Medical Sciences, Tehran 141556447, Iran; 3 Health Economics & Management, Institute of Economic Research, Lund University, Lund 22007, Sweden; 4 Health Insurance Office, Ministry of Cooperatives-Labor and Social Welfare, Tehran 1457994861, Iran; E-Mail: rezahp1979@gmail.com; 5 Institute for Global Health, University College London, London WC1N 1EH, UK; E-Mail: h.haghparast-bidgoli@ucl.ac.uk; 6 Department of Economics, Lund University, Lund 22363, Sweden

**Keywords:** dentists, disparity, Gaswirth index of disparity, relative index of inequality, ecological, Iran

## Abstract

During past decades, the number of dentists has continuously increased in Iran. Beside the quantity, the distribution of dentists affects the oral health status of population. The current study aimed to assess the pure and social disparities in distribution of dentists across the provinces in Iran in 2009. Data on provinces’ characteristics, including population and social situation, were obtained from multiple sources. The disparity measures (including Gini coefficient, index of dissimilarity, Gaswirth index of disparity and relative index of inequality (RII)) and pairwise correlations were used to evaluate the pure and social disparities in the number of dentists in Iran. On average, there were 28 dentists per 100,000 population in the country. There were substantial pure disparities in the distribution of dentists across the provinces in Iran. The unadjusted and adjusted RII values were 3.82 and 2.13, respectively; indicating area social disparity in favor of people in better-off provinces. There were strong positive correlations between density of dentists and better social rank. It is suggested that the results of this study should be considered in conducting plans for redistribution of dentists in the country. In addition, further analyses are needed to explain these disparities.

## 1. Introduction

Oral health is considered an important part of populations’ health and wellbeing [1]. However, while prevalence of oral disease is increasing in many low- and middle income countries [1], in many countries less attention has been given to oral health [2]. A recent literature review showed that dental caries as a major oral health problem is markedly increasing worldwide [3]. In addition, the global trend in lifestyles towards increased consumption of sugar and alcohol as well as smoking suggest that burden of oral disease will persist in the future in many countries [4].

Beside lifestyle factors, the social rank (SR) of individuals and regions are significantly associated with oral health status [5,6,7,8,9]. Additionally, there are in general social inequalities in access and utilization of dental services [10,11,12,13]. In other words, people with a lower SR not only have poorer oral health status, but they may also have lower access to oral health care resources. These issues brought the WHO to encourage countries to incorporate oral health as an integral part of policies for prevention of non-communicable diseases and to promote the accessibility and availability of oral health services especially for poor and disadvantaged populations [1]. 

In Iran, dental care is mainly provided by the private sector in cities and by the public sector in rural areas. Since 1997, oral health has been integrated into the primary health care (PHC) network within Iran. Following this, dental care is delivered in four levels in the country, from primary prevention in rural areas to specialists’ care in the cities [14]. 

Moreover, during past decades, the number of dentists has increased substantially in the country from 3,500 in 1990 to 11,000 in 2,000 [14]. Based on latest available data from the Iran Medical Council (IMC) [15], more than 20,000 dentists are currently practicing in the public and private sectors in Iran (only 20% of dentists work in public sector and remaining 80% have private practices [13]). In 2012 approximately 1,400 students were admitted to dental schools across the country, a figure which rose steadily through the preceding decade, as demonstrated by the increase in dental schools, which has risen from 18 in 2000 to 44 in 2012 [14,16]. 

It is well-established that increasing the number of health inputs, including dentists, does not necessarily result in improved health, but how they are distributed is also a determinant factor [17]. Actually, access to health care and distribution of resources within health sector is considered as one of the social determinants of health [18,19]. Although studies in Iran have examined the association between demographic and SR with population’s oral health status [20,21,22] and utilization of dental services [23], little attention has been paid to the distribution of dentists across the provinces in Iran.

To fill this gap of knowledge, this study examined availability of dentists in Iran using various disparity measures which evaluate different aspects of disparity. We focused on following two research questions: How were the dentists distributed across the provinces in 2009? And was this distribution associated with the provinces’ SR? These questions are among policy interests as equity in access to health care is a common goal of policy-makers in all countries. Moreover, response to these questions is relevant for health resource (here dentists) allocation policies across the provinces in Iran.

## 2. Method and Materials

### 2.1. Study Setting

Iran, a lower-middle-income country, is located in the Eastern Mediterranean Region with an area of 1,648,000 km sq. Based on the census data in 2011, a population of about 75 million people are living in 31 provinces in Iran [24]. 

### 2.2. Data Sources and Variables

The data on the distribution of population at the province level were obtained from the National Organization for Civil Registration [25]. The data on the provinces’ SR were obtained from the Statistical Centre of Iran and the President Deputy of Strategic Planning and Control [24,26]. The data on the number of dentists practicing in public and private sectors in each province were gathered from the Iran Medical Council [15]. In the current study, the number of dentists per 100,000 people (DPR) was used as the indicator for availability of dental care resources for the population in each province.

### 2.3. Disparity Measures

We evaluated two types of disparities in the current study: pure and social. In pure disparity, we examined how dentists were distributed across the provinces in Iran regardless the provinces’ socioeconomic characteristics. Then, we examined whether social disparity among the provinces can explain the distribution of dentists across the provinces. Three different measures were used to examine the pure disparity: Gini coefficient, index of dissimilarity (ID) and Gaswirth index of disparity (GID). These measures respond to different research questions. The relative index of inequality (RII) was used to measure the social disparity. These measures are defined as follows: 

#### 2.3.1. Pure Disparities

##### 2.3.1.1. Lorenz Curve and Gini Coefficient

These are commonly used in assessing the pure disparity in distribution of health care resources [17,27,28,29]. Lorenz curve is used to compare distribution of specific health variable with perfect equality (diagonal line). This curve plots the cumulative share of population ranked by health variable, in an increasing order, against the cumulative share of health variable. The further the distance from diagonal line implies the greater degree of disparity. The Gini coefficient is equal to twice the area between the Lorenz curve and diagonal. Its value ranges from 0 (perfect equality) to one (maximum possible inequality). We used the formula proposed by Brown [30] to calculate Gini coefficient:

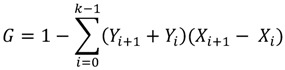
(1)
where *G* is Gini coefficient; *Y_i_* is cumulative share of dentists in ith province; *X_i_* is cumulative share of population (ranked by DPR) in ith province; and *k* is the total number of provinces. This measure takes into account the distribution of health variable in the entire population. 

##### 2.3.1.2. Index of Dissimilarity (ID)

This index estimates the proportion of total health variable, which would need to be redistributed across provinces to achieve a situation of perfect equality [31] and is calculated through following formula:

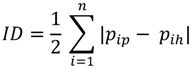
(2)
where *p_ip_* is ith province’s share of population; *p_ih_* is ith province’s share of health variable; and *n* is the total number of provinces.

##### 2.3.1.3. Gaswirth Index of Disparity (GID)

This index measures relative increase in health variable to bring the entire population to the level received by reference group [32]. It is recommended that the rate of the best group should be used as reference rate. It is calculated in two steps: first, the fraction of entire population that is under-served relative to the best group is calculated as follows:

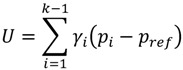
(3)
where γ*_i_* is ith province’s share of population; *p_i_* is a measure of health variable in ith province; and *k* is total number of provinces. 

Then the *GID* is calculated as *GID*=*U/p*. The p is the mean of health variable (dentists, here) in the entire population. While *ID* estimates that how available dentists should be redistributed (among geographical units or provinces) to achieve an equal distribution (equal DPR across provinces), the *GID* estimates that how many new dentists should be added to current number of dentists in each province to reach the DPR equal to level of province with the highest DPR. 

#### 2.3.2. Social Disparities

##### Relative Index of Inequality (RII)

It is a regression-based method to measure the social disparities in health. RII takes into account the population distribution across social groups [28]. After determining the relative position of the population in the provinces ranked by socioeconomic position, the number of dentists in the provinces was regressed on these relative ranks using negative binomial regression with a robust variance. In this case, an RII value greater (lesser) than 1 show that dentists are more available in the provinces with higher (lower) SR. To account for effect of demographic confounders (age and gender), the adjusted RII was also calculated by including the proportion of female in the population, proportion of people younger than 15 years old and proportion of people older than 65 years old as covariates in our regression. The reason for including these covariates in the analysis was that the previous studies showed that age and gender are significantly associated with demand for dental services in the country [23,33].

### 2.4. Data Analysis

In the current study, the geographic unit of analysis was 30 provinces in Iran (as there were 30 provinces in the country in 2009 and Tehran province was split in two provinces later). To rank the provinces, we used the Human Development Index (HDI), average total expenditures per head (TXH) and average non-food expenditure per head (NFXH). Moreover, the unemployment rate for people of 10 years and older and urbanization rate were used as proxies of SR in correlation analysis. To account for economies of scale in household expenditures in calculating the TXH and NFXH, the household’s total and non-food expenditures were divided on the equivalent scaled household size by raising household size to the power 0.56 [34]. To calculate the social disparities, the HDI was used as main variable and TXH and NFXH used in the sensitivity analysis. Furthermore, in another sensitivity analysis, Tehran province was excluded from the analysis to examine the pure and social disparities across the remaining provinces. The reason for this was that Tehran has special situation as the capital of the country and being the centre of economic, social and political activities. The pairwise correlations between DPR and each of the social ranking variables were calculated to examine if there is any linear relationship between the distribution of dentists and the provinces’ SR. Data were analyzed using Excel 2010 and STATA version 11. 

## 3. Results

**Figure 1:**
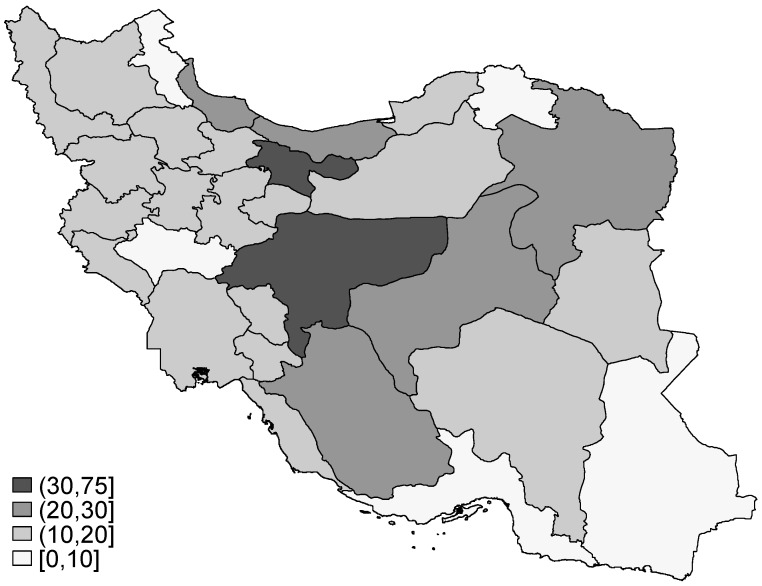
Distribution of dentists per 100,000 population across the provinces in Iran, 2009.

**Figure 2:**
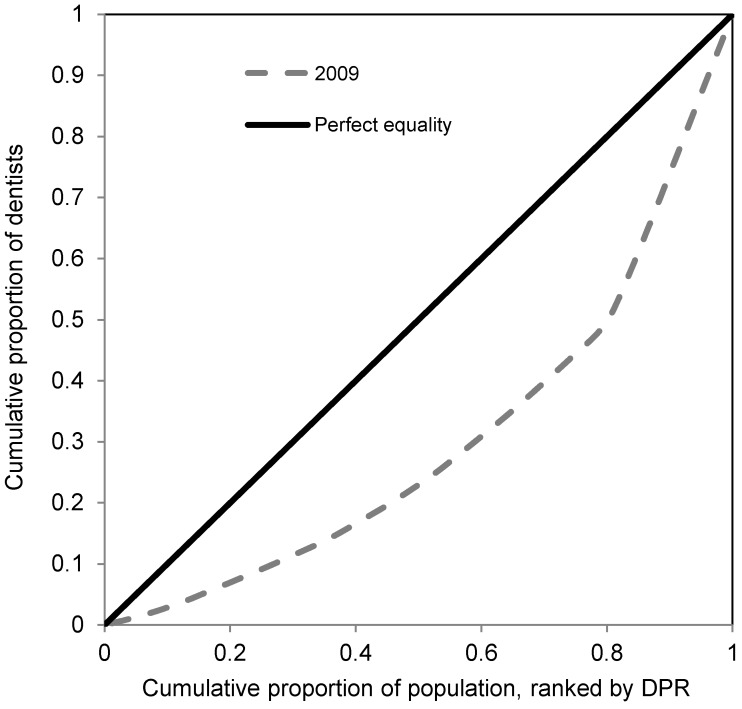
Lorenz curve of the distribution of dentists in Iran, 2009.

Figure 1 shows how dentists were distributed across the provinces in Iran in 2009. On average, there were 28 dentists per 100,000 population in the country (range 7–71). There were substantial differences across the country and value of DPR was 11 times higher for Tehran (with the highest DPR) compared to Northern Khorasan (with the lowest DPR). Only three out of 30 provinces had a DPR equal or greater than the country’s average (*i.e*., Yazd, Isfahan and Tehran). As it can be seen from Figure 1, most provinces (60%) had a DPR value of 10 to 20. Table 1 shows the pure and between-area social disparity measures in the distribution of dentists across the country. The Gini coefficient was equal to 0.39 which implies substantial disparity across the country. The Lorenz curve corresponded to this Gini coefficient is been shown in Figure 2. 

**Table 1:** Pure and between area-socioeconomic disparity measures in the distribution of dentists in Iran, 2009.

Disparity measure	Total sample	Sample excluding Tehran
Gini coefficient	0.39	0.23
Index of Dissimilarity (%)	30.48	17.64
Gaswirth index of disparity	1.55	0.84
Relative index of inequality	3.82 (1.91–7.64)	2.12 (1.39–3.24)
Adjusted relative index of inequality *	2.13 (1.01–4.48)	1.48 (0.90–2.43)

* Adjusted for female proportion in population, population younger than 15 years old and population older than 65 years old.

The value of the ID implies that in order to have an equal distribution, 6,193 of current dentists should be redistributed in the country. On the other hand, results of GID show that about 43% of the populations in the country are under-served when compared with Tehran’s population and a number of 31,583 new dentists (GID × current number of dentists) are needed in the country to bring the DPR to the level available for Tehran province. Both unadjusted and adjusted RII (ranked by HDI) showed that there were social disparities in the distribution of dentists in favor of better-off provinces. The same results were obtained when THX was used to rank the provinces in the sensitivity analysis. However, when we used NFXH for ranking, the adjusted RII was not statistically significant. Although excluding Tehran province from the sample decreased the magnitude of pure and social disparities, significant disparities remained in the distribution of dentists across the provinces. 

Table 2 shows that how dentists should be distributed in each province based on the results of ID and GID. Based on the results from ID, the main redistribution should happen from Tehran province where approximately 61% of the current dentists within this province should be redistributed to other provinces. On the other hand, if government wants to increase the availability of dentists in all provinces to the level of Tehran province (*i.e.*, 71 dentists per 100,000 population), then the lowest and the highest number of new dentists are needed in Ilam and Khorasan Razavi provinces, respectively.

**Table 2:** The required changes in the distribution of dentists across the country to reach perfect equality.

Province	Number of dentists	Changes based on index of dissimilarity	Increases based on gaswirth index of disparity
Ardebil	103	+251	+802
Bushehr	111	+146	+545
Chaharmahal Bakhtiari	105	+144	+532
East Azerbaijan	717	+320	+1,932
Fars	1,156	+91	+2,031
Gilan	534	+149	+1,211
Golestan	270	+200	+931
Hamedan	252	+237	+998
Hormozgan	129	+280	+917
Ilam	103	+54	+299
Isfahan	1,536	−233	+1,793
Kerman	459	+310	+1,506
Kermanshah	245	+296	+1,138
Khuzestan	530	+715	+2,649
Kohkiluye & Boyerahmad	118	+67	+354
Kordestan	182	+233	+879
Lorestan	169	+328	+1,100
Markazi	169	+217	+817
Mazandaran	745	+89	+1,386
Northern Khorasan	57	+180	+547
Qazvin	197	+131	+641
Qom	141	+161	+630
Khorasan Razavi	1,355	+268	+2,792
Semnan	104	+64	+326
Sistan & Baluchestan	188	+530	+1,647
Southern Khorasan	67	+117	+404
Tehran	9,778	−5,950	0
West Azerbaijan	386	+447	+1,743
Yazd	296	−9	+436
Zanjan	113	+165	+597

Table 3 presents the pairwise correlations between DPR and SR across the provinces in Iran. It can be seen that there were strong positive correlations between the density of dentists and the provinces’ SR. Moreover, the provinces with higher proportion of population living in urban areas had higher density of dentists. The association between unemployment rate and DPR was small and statistically non-significant.

**Table 3:** Pairwise correlations between dentists to population ratio and various measures of social rank.

Variable	DPR	HDI	TXH	NFXH	Urbanization
DPR					
HDI	0.61 ***				
TXH	0.64 ***	0.62 ***			
NFXH	0.70 ***	0.54 **	0.78 ***		
Urbanization	0.57 ***	0.69 ***	0.52 **	0.54 ***	
Unemployment	0.02	−0.09	0.22	0.10	0.02

***, ** and *: 0.001, 0.01 and 0.05 significant level, respectively. DPR: Dentist to population ratio. HDI: Human Development Index. TXH: Total expenditures per head. NFXH: Non-food expenditure per head.

## 4. Discussion

The current study is the first national study that assessed the availability of dentists across the provinces in Iran. The results showed that while availability of dentists in Iran is higher than global average (22 per 100,000 population [35]), there were substantial pure and social disparities in the distribution of dentists across the country, and generally dentists were located in the provinces with better SR. To achieve an equal pure distribution of dentists across the country, about three out of 10 dentists should be redistributed from the over-served provinces to the under-served ones. 

The previous national and international studies reported that people with lower SR had poorer oral health than people with higher SR [5,6,7,8,9,20,21,22,36]. The results of our study imply that the lower availability of dentists for these people may partly explain the disparity in oral health. 

There are some potential explanations for the pure and social disparities in the distribution of dentists in Iran. Firstly, about 80% of dentists are working in private sector [14] and dental services are generally provided with high copayments by users. Since people with higher SR have higher capacity to pay and/or higher knowledge about the importance of oral health, then they have potentially higher demand for dental services than their counterpart with poor SR [37,38,39]. Secondly, as it was shown by a recent study in Iran [40], better employment and social opportunities are determinant factors in decision-making for their career among dentists. It seems that the provinces with better SR offer better opportunities for dentists and hence dentists are more motivated to practice at these provinces. Thirdly, several studies have demonstrated that new physicians are more likely to practice in the region (or states) where they have finished their medical school or residency training [41,42]. Considering the fact that 20% of dental schools are located in two provinces with the highest DPR and better social position (*i.e*., Tehran and Isfahan), this can partly explain the pure and social disparities in the distribution of dentists in the country. Of course, it should be noted that graduated dentists cannot practice in metropolitan areas like Tehran and Isfahan immediately after graduation and they have to work for a few years (3–7 years depending on deprivation and remoteness of the city) in smaller cities to qualify to practice in larger cities. 

The unequal distribution of dentists has also been reported in the other countries. Kruger *et al.* [43] reported significant geographic disparities in private dental practice in Western Australia with a higher density in the regions with better SR. In another study in Japan, a Gini coefficient equal to 0.255 was reported for the distribution of dentists in year 2000 [44]. Moreover, the WHO reported a range of less than 0.5 to 40 dentists per 100,000 population around world implying substantial disparity in the distribution of dentist at a global level [35]. Compared to other health resources, distribution of dentists are more unequal across the provinces in Iran. For example, previous studies have shown that the Gini coefficient for specialist physicians, nurses, active hospital beds, rural health houses and pre-hospital trauma care facilities were equal or less than 0.20 in Iran [17,45,46]. 

The results of the current study should be interpreted in light of some limitations. Firstly, potential incompleteness and measurement errors in the registry data utilized in this study may bias the results. If number of dentists were underestimated (overestimated) for the provinces with lower SR, then there is overestimation (underestimation) in our social disparity. Secondly, the current study is an ecological study at province level. It means that the observed pure and social disparities are between-provinces and it is not valid for within-province variations. Hence, generalizability of the results to smaller geographic units or individuals is limited. Thirdly, prevalence of dental caries and periodontal diseases are among the main determinants of need for the dental services which in turn is a determinant factor in the distribution of dentists. As it was shown in the previous studies [47,48], there are geographic disparities in the distribution of these disorders, implying the need for controlling these factors when examining the distribution of dentists. However, the lack of data on these disorders confined us to control for them. It should be noted that the previous studies in Iran [20,21,22] have shown that people with lower SR have higher need of dental services. This implies that the disparity of dentists may be more profound than what was found in our study. Fourthly, this is a descriptive cross-sectional study which implies that any causal inference from the results should be avoided. 

Despite these limitations, the findings of this study provide valuable information for health care policy makers. It is suggested that the current policies should be reviewed and some new policies developed to narrow the pure and social disparities in the distribution of dentists in the country. Some potential policies include: allocating a number of training positions at universities for students from the provinces with lower SR to practice in their home provinces after graduation, offering economics incentives for dentists if they practice in the provinces with lower SR and remote areas, promoting coverage of dental services by health insurance system (in terms of population, services and costs) in the country. Promoting insurance coverage may also decrease income uncertainty for the dentists in these provinces. 

## 5. Conclusions

This study demonstrated that there are substantial pure and social disparities in the distribution of dentists across the provinces in Iran. Generally dentists are located in the provinces with better SR. It is suggested that the results of this study be considered in making decisions on the dental service system in Iran including the dentistry education and health insurance system. In addition, further analyses are needed to explain these pure and social disparities in the distribution of dentists in Iran.
